# The mediating role of recreational flow experience in the relationship between adventure behavior seeking and event satisfaction among participants in outdoor leisure activities

**DOI:** 10.1186/s40359-026-04254-6

**Published:** 2026-02-28

**Authors:** Sertaç Erciş, Davut Budak

**Affiliations:** 1 Department of Recreation, Faculty of Sports Sciences, Atatürk University, Erzurum, Türkiye; 2 Department of Sports Management, Faculty of Sports Sciences, Atatürk University, Erzurum, Türkiye

**Keywords:** Adventure behavior, Recreational flow experience, Event satisfaction, Leisure activities

## Abstract

**Objectives:**

Outdoor leisure activities involve individuals engaging in high levels of physical and psychological interaction, where the sense of satisfaction plays a crucial role. In this context, examining the psychological processes underlying the relationship between adventure behavior seeking and event satisfaction is important for the design of outdoor activities and the enhancement of participant experience. Building on this, the aim of the study is to investigate the mediating role of recreational flow experience in the relationship between adventure behavior seeking and event satisfaction among individuals participating in outdoor leisure activities.

**Methods:**

This study involved 501 participants engaged in outdoor recreational activities, selected through convenience sampling. Participants completed the Adventure Behavior Seeking Scale, Recreational Flow Experience Scale, and Event Satisfaction Scale. Data were analyzed using the PROCESS macro for SPSS, with the proposed mediation model tested via Model 4.

**Results:**

The mediation analysis using Model 4 of the PROCESS macro revealed a partial mediating effect of recreational flow experience in the relationship between adventure behavior seeking and event satisfaction. Structural equation modeling indicated that adventure behavior seeking had a strong and statistically significant positive effect on recreational flow experience (β = 0.75, *p* < 0.001). Recreational flow experience, in turn, significantly predicted event satisfaction (β = 0.60, *p* < 0.001). Additionally, the direct effect of adventure behavior seeking on event satisfaction was significant (β = 0.21, *p* = 0.018), indicating partial mediation. The indirect effect via recreational flow experience was also significant (β = 0.68, *p* = 0.001).

**Conclusions:**

These results suggest that individuals with higher adventure behavior seeking tendencies experience greater satisfaction in outdoor events both directly and indirectly through enhanced recreational flow experiences during participation. By examining both the direct and mediated effects of adventure behavior seeking on event satisfaction, this study contributes to a deeper understanding of the psychological mechanisms that enhance participant experience in outdoor leisure activities.

**Supplementary Information:**

The online version contains supplementary material available at 10.1186/s40359-026-04254-6.

## Background

Outdoor and adventure-based leisure activities have increasingly drawn the attention of both researchers and individuals, not only for their physical benefit but also for their positive impact on mental health and social connection. As today’s life becomes more sedentary, digitally dominated, and routine-driven, many individuals are seeking opportunities in nature to rediscover themselves, overcome challenges, and engage in meaningful experiences [1]. These activities allow participants to escape everyday stressors and immerse themselves in stimulating physical and psychological experiences, contributing to overall well-being and community connection. Moreover, engagement in these environments encourages adaptive coping strategies, allowing individuals to develop resilience in facing both physical and emotional challenges. Participation often facilitates social bonding through shared experiences and collaborative problem-solving [2]. Understanding the motivational factors behind such engagement can be effectively approached through sensation seeking theory (SST) as proposed by Zuckerman [3]. The theory suggests that individuals differ in their inherent desire for novelty and intensity, with high sensation-seekers pursuing unpredictable and risky activities to reach optimal arousal. Those with high sensation-seeking tendencies are especially inclined to pursue activities that involve unpredictability and risk reaching an optimal arousal state. Adventure activities marked by elements of danger, uncertainty, and interaction with raw natural environments often fulfill this need [1]. Beyond thrill-seeking impulses, participation is associated with enhanced self-confidence, emotional regulation, and psychological development [4].

Recent perspectives increasingly characterize adventure behavior as a multifaceted and psychologically rich phenomenon that transcends mere physical challenge. Rather than viewing adventure solely in terms of risk-taking, Próchniak [5] emphasizes its role in processes of self-exploration and the search for existential meaning. This broader perspective highlights that adventure engagement can act as a catalyst for lifelong learning, promoting critical reflection and personal insight. Furthermore, the unpredictability of outdoor settings can stimulate creativity and problem-solving, which may transfer to everyday life. Adventure behavior emerges through a dynamic interplay of personal traits, situational factors, and underlying motivational drives. Thus, outdoor adventure is both physical engagement and deeply transformative experience, offering opportunities for psychological growth and identity development [6]. Within this framework, flow theory [7] offers a valuable approach to understanding the psychological underpinnings of satisfaction in intense, high-engagement activities. Flow is defined as a state of optimal experience in which individuals become fully absorbed in a task, experiencing deep focus, intrinsic motivation, and a diminished sense of self. Flow emerges when there is a balance between challenge and skill, and outdoor recreation’s dynamic, unpredictable nature supports flow experiences [8–10].

Individuals with high sensation-seeking tendencies are particularly inclined to experience the flow in adventure-based settings, as such environments offer the novelty, challenge, and intensity they seek. Once achieved, the flow state can significantly enhance emotional and psychological outcomes, contributing to greater satisfaction, subjective well-being, and a meaningful sense of personal achievement [11, 12]. Flow thus mediates the influence of adventure behavior on positive experiential outcomes. Empirical studies show that high-risk, skill-based activities such as climbing, white-water kayaking, and skydiving provide optimal conditions for flow and personal development [1]. Overall, outdoor adventure experiences satisfy immediate psychological needs while contributing to broader personal and social growth, shaping resilient, self-aware, and socially connected individuals.

## The present study

Grounded in both theoretical insights and empirical evidence, the present study seeks to examine the relationship between adventure behavior seeking and event satisfaction, with particular emphasis on the mediating role of recreational flow experience. Despite increasing research on adventure participation, there has been limited attention on how recreational flow specifically functions as a psychological mechanism linking adventure-seeking behavior to satisfaction. This lack of focus is important because without understanding this mechanism, interventions and program designs may fail to optimize participant engagement and psychological benefits, and the theoretical understanding of adventure behavior remains incomplete. By investigating how flow links adventure-seeking motivations with satisfaction derived from participation in an outdoor recreational event, this research aims to contribute to a more nuanced understanding of affective and motivational processes in nature-based recreation. By addressing this gap, the study uniquely contributes to existing research by examining flow as a mediator in diverse outdoor contexts and among varied participant groups, including adults involved in structured outdoor adventure activities, youth with disabilities, and individuals from underrepresented geographic or cultural backgrounds. Moreover, the study enriches the conceptualization of adventure behavior as a measurable construct in leisure research [13] offering broader implications for personality research, motivation studies, and well-being promotion in outdoor experiential contexts.

The present study targets adult participants engaged in organized outdoor adventure activities such as climbing, mountain biking, and kayaking, with a focus on structured recreational events in natural settings. These participants comprise a growing demographic driven by desire for psychological well-being, social connection, and personal challenge [14]. Participants of such events have been shown to seek not only physical activity but also community belonging and emotional support through shared outdoor experiences [15]. This sample is particularly relevant as recent studies emphasize the physical and mental health benefits associated with high-engagement outdoor recreation, particularly when it occurs in forested or remote natural environments [16]. Additionally, including diverse participant profiles, such as youth with disabilities or those from underserved backgrounds, highlights the need for inclusive and adaptive programming, which may provide insights into how context shapes adventure experience [17]. Furthermore, examining participants from particular geographic and cultural contexts, including rural or developing regions, provides a deeper understanding of how adventure infrastructure fosters both personal growth and community well-being [18]. As such, the chosen population offers both theoretical relevance and practical implications for adventure recreation research.

Despite these efforts, few studies have empirically tested the mediating role of flow across culturally and environmentally diverse contexts, leaving important questions about generalizability unanswered [19]. By targeting these gaps, the present study seeks to elucidate how personal traits and situational factors collectively influence the quality and outcomes of adventure experiences. Addressing these underexplored mechanisms not only advances theoretical understanding of adventure behavior and flow but also provides actionable insights for designing outdoor programs that optimize participant satisfaction and well-being across diverse populations.

Grounded in this rationale, the primary aim of this study is to investigate the relationship between adventure behavior seeking and event satisfaction, with a particular emphasis on the mediating role of flow experience in an outdoor recreational context. This study integrates sensation seeking theory and flow theory by proposing that individual differences in adventure behavior seeking (rooted in SST) influence the likelihood of achieving optimal psychological states (flow), which in turn drive experiential satisfaction. Conceptually, SST explains why individuals are motivated to pursue high-arousal, novel, and challenging activities, while flow theory clarifies how engagement in such activities produces immersive experiences that enhance satisfaction [3]. Together, these frameworks provide a coherent explanation for the mediating mechanism linking adventure behavior seeking to event satisfaction, highlighting the compatibility of personality-driven motivation and experiential processes in shaping outdoor leisure outcomes. Drawing on SST [3] and flow theory [7] the study explores how personality-based motivation for adventure translates into affective satisfaction through optimal psychological states. Previous studies have indicated that individuals with high sensation-seeking tendencies are more likely to engage in high-risk recreational activities that foster immersive and challenging experiences [1]. These experiences were found to enhance flow and psychological benefits such as self-confidence and well-being [16]. Additionally, flow was shown to be a significant mediator between intrinsic motivation and subjective satisfaction in physically demanding recreational settings [14]. Despite this body of research, there is limited empirical evidence examining how flow specifically mediates the relationship between adventure-seeking tendencies and event satisfaction across diverse outdoor settings. By addressing this gap, the present study highlights the unique contribution of flow as a psychological mechanism linking individual motivations to experiential outcomes. This focus provides practical insights for designing outdoor programs that enhance participant engagement and well-being. Based on this theoretical and empirical foundation, the following hypotheses are proposed:

- Hypotheses 1 Adventure behavior seeking positively affects event satisfaction.
- Hypotheses 2 Adventure behavior seeking positively affects recreational flow experience.
- Hypotheses 3 Recreational flow experience positively affects event satisfaction.
- Hypotheses 4 Recreational flow experience mediates the relationship between adventure behavior seeking and event satisfaction.

## Methods

### Participants

This study employed a cross-sectional quantitative design aimed at examining the relationships among key variables within a specific timeframe, utilizing data collected from participants engaged in relevant activities. The sample consisted of 501 individuals who participated in outdoor recreational activities and were recruited through convenience sampling between April and June 2025 in various cities across Turkey. The sample included 233 females (46.5%) and 268 males (53.5%). Participants’ ages ranged from 18 to 50 years (Mean = 23.02, SD = 6.58). Regarding education level, 345 participants (68.9%) had completed high school, while 156 (31.1%) held university or postgraduate degrees. In terms of employment status, 316 (63.1%) were unemployed, 117 (23.4%) worked part-time, and 68 (13.6%) were employed full-time. Participants’ weekly leisure time was distributed as follows: 96 (19.2%) engaged in 1–5 h, 150 (29.9%) in 6–10 h, 120 (24.0%) in 11–15 h, and 135 (26.9%) in 16 or more hours. Regarding the type of outdoor activities, 52 participants (10.4%) preferred air-based, 93 (18.6%) water-based, and 356 (71.1%) land-based activities. Annual frequency of participation showed that 389 (77.6%) engaged 1–5 times, 52 (10.4%) 6–10 times, and 60 (12.0%) 11 or more times.

### Data collection and ethical procedure

Ethical approval for the study was granted by the corresponding author's university ethics committee (Date: 26/03/2025; Decision No: E-70400699-2500104950) before the commencement of data collection. The data were obtained through an online survey distributed across various social media platforms such as Facebook, WhatsApp, and Twitter. Prospective participants received detailed written explanations concerning the study objectives, scientific background, and confidentiality of their responses. Inclusion criteria required participants to be aged 18 or older, free from medical conditions restricting physical activity, and able to provide informed consent. On average, respondents completed the survey within eight to ten minutes. From the total 526 respondents, 501 met the eligibility criteria and were included in the final analysis. Twenty-five participants were excluded due to incomplete responses, failure to meet the inclusion criteria (e.g., being under 18 years old or having medical conditions restricting physical activity), or presenting inconsistent or illogical answers identified during data screening.

### Measurements

#### Adventure behavior seeking scale

The scale, originally created by Próchniak [5] and subsequently adapted to Turkish culture by Güngörmüş et al. [13], assesses individuals’ tendencies toward adventure-seeking behavior through two subscales: “water threats” (e.g., I swim far from the shore) and “weather conditions” (e.g., I go hiking even if the weather is cold or windy). The scale consists of seven items in total. Higher scores indicate a stronger propensity for adventure-related experiences. For this study, the scale demonstrated acceptable internal consistency, with a Cronbach’s alpha coefficient of 0.72.

For this study, the ABSS was used as a single construct. This approach is supported by previous research showing that, although the scale includes two subscales, it can also be treated as a unidimensional measure of adventure-seeking tendencies through second-order factor analysis. To confirm unidimensionality in the current sample, a Confirmatory Factor Analysis (CFA) was conducted. The CFA results indicated acceptable model fit (χ²/df = 5.99, CFI = 0.91, GFI = 0.95, RMSEA = 0.08, NFI = 0.90, SRMR = 0.07), supporting the appropriateness of using the scale as a single construct. In addition, standardized factor loadings ranged from 0.57 to 0.75, indicating that all items made a satisfactory contribution to the latent construct.

#### Recreational flow experience scale

The scale developed by Ayhan et al. [8] assesses flow experience during recreational activities through nine items. Responses are provided on a 7-point Likert scale ranging from 1 (strongly disagree) to 7 (strongly agree). This unidimensional scale captures the overall flow experience. An example item is: “I feel that I have a positive experience during the activity.” In the present study, the scale exhibited excellent internal consistency, with a Cronbach’s alpha coefficient of 0.96, reflecting high reliability.

#### Event satisfaction scale

The event satisfaction measure was originally developed by Oliver [20] to capture individuals’ overall subjective evaluation of their decision to participate in an event, reflecting their satisfaction with the participation experience. This scale was later adapted by Funk et al. [21] to assess satisfaction in the context of mass participation sport events. In the present study, the scale was further modified to specifically capture satisfaction in outdoor leisure activities, with items revised to align with the unique features and experiences of these settings. The final version evaluates satisfaction with participation in outdoor leisure activities, and responses are recorded on a 7-point Likert scale ranging from 1 (strongly disagree) to 7 (strongly agree). In addition, the questionnaire was translated into Turkish and then independently back-translated into English by bilingual experts to ensure that the meaning of each item was accurately preserved. A native English speaker reviewed both versions to confirm clarity and conceptual consistency, thereby supporting the reliability and validity of the measurement tool. The internal consistency of the scale was found to be high in the current study, with a Cronbach’s alpha coefficient of 0.91.

### Data analysis

Before conducting the main analyses, the distribution of the variables was assessed to verify the assumption of normality by examining skewness and kurtosis coefficients, with values falling within the ± 2 range interpreted as indicative of approximate normality, an acceptable criterion for parametric analyses [22, 23]. Following this, descriptive statistics, including means and standard deviations, were calculated to provide a general profile of the data. To explore the correlations among the study variables, Pearson’s product-moment correlation analysis was employed. This method is well-suited for detecting linear associations between continuous variables and is widely adopted in behavioral sciences research [24]. The proposed mediation model was tested using Model 4 of the PROCESS macro for SPSS, developed by [25]. This analytical approach applies bootstrapping, a non-parametric resampling technique, with 5000 iterations to generate confidence intervals for the estimation of indirect effects. Complementing the bootstrapping results, the Sobel Z test [26] was conducted to further assess the significance of the mediating effect. The internal consistency of the measurement instruments was evaluated using Cronbach’s alpha coefficients. In line with established guidelines, alpha values equal to or above 0.70 were considered acceptable indicators of internal consistency [27]. Additionally, convergent validity was assessed through the calculation of Average Variance Extracted (AVE) and Composite Reliability (CR) value s.

### Findings

#### Descriptives, normality distribution, internal consistency, and convergent validity

The study sample consisted of 501 individuals, including 233 females (46.5%) and 268 males (53.5%). In terms of education, the majority had completed high school (68.9%), while the remaining held university or postgraduate degrees. Regarding employment status, 63.1% of participants were unemployed, 23.4% worked part-time, and 13.6% were employed full-time. Land-based activities were the most commonly practiced (71.1%), followed by water-based (18.6%) and air-based activities (10.4%). Participants’ preferences for activity-related risk varied, with nearly half (48.5%) favoring moderate-risk options. Annual participation frequency was predominantly one to five times per year (77.6%), with fewer participants attending six to ten times (10.4%) or eleven or more times (12.0%). Most individuals reported engaging in outdoor activities with friends (74.5%), while others participated alone or with family members. These descriptive statistics are summarized in Table 1.

**Table 1:** Demographic and leisure characteristics of participants

Variables	Categories	Frequency	%
Gender	Female	233	46.5
	Male	268	53.5
Education Status	High school	345	68.9
	University / Postgraduate	156	31.1
Employment Status	Unemployed	316	63.1
	Part-time	117	23.4
	Full-time	68	13.6
Type of Outdoor Activity	Air-based	52	10.4
	Water-based	93	18.6
	Land-based	356	71.1
Preferred Risk Level	Low	211	42.1
	Medium	243	48.5
	High	47	9.4
Annual Activity Frequency	1–5 times	389	77.6
	6–10 times	52	10.4
	11 or more times	60	12.0
Participation Style	Alone	84	16.8
	With friends	373	74.5
	With family	44	8.8

Participants reported moderate levels of perceived behavioral safety (ABSS; Mean = 2.51, SD = 0.65), accompanied by relatively high levels of risk-focused engagement (RFES; Mean = 5.32, SD = 1.47) and emotional satisfaction in sport (ESS; Mean = 5.49, SD = 1.52), as indicated in Table 2. Skewness and kurtosis values for all variables fell within acceptable ranges (± 2), supporting the assumption of approximate normality [22, 23]. Internal consistency, as indicated by Cronbach’s alpha coefficients, ranged from 0.72 to 0.96, surpassing the recommended minimum threshold of 0.70 [27]. Furthermore, convergent validity was supported, with Average Variance Extracted (AVE) values exceeding 0.50 and Composite Reliability (CR) values surpassing 0.70 across all constructs [28]. In addition, correlation analysis indicated significant positive relationships among the three variables, with the strongest observed between recreational flow and event satisfaction (*r* = 0.61), followed by a notable association between adventure behavior seeking and recreational flow.

**Table 2:** Descriptive statistics, reliability and convergent validity indicators

Constructs	ABSS	RFES	ESS
ABSS			
RFES	0.33**		
ESS	0.28**	0.61**	
M	2.51	5.32	5.49
SD	0.65	1.47	1.52
Skewness	0.77	-0.78	-0.82
Kurtosis	-0.41	0.02	0.02
Cronbach’s Alpha	0.72	0.96	0.91
AVE	0.64	0.76	0.90
CR	0.84	0.97	0.96

*ABSS* Adventure Behavior Seeking Scale, *RFES* Recreation Flow Experince Scale, *ESS* Event Satisfaction Scale, *M* Mean, *SD* Standard deviation, *CR* Composite reliability, *AVE* Average variance extracted

**p < 0.01

#### Conceptual model of research analysis results

The mediation analysis results showed a partial mediating effect of recreational flow experience in the correlation between adventure behavior seeking and event satisfaction. Structural equation modeling results indicated that adventure behavior seeking had a strong and statistically significant positive effect on recreational flow experience (β = 0.75, *p* < 0.001). In turn, flow experience was found to significantly predict event satisfaction (β = 0.60, *p* < 0.001). In addition to this indirect pathway, the direct effect of adventure behavior seeking on event satisfaction was determined as statistically significant (β = 0.21, *p* < 0.018), indicating a partial mediation. The indirect effect of adventure behavior seeking satisfaction through flow was also substantial and significant (β = 0.68, *p* < 0.001, Table 3).

**Table 3:** Mediating results of structural equation model

Variables	Coefficient (β)	Standard Error	t value	*p* value
ABSS →RFES(a)	0.75	0.09	7.80	0.000
RFES→ESS (b)	0.60	0.04	15.73	0.000
ABSS→ ESS (c’)	0.21	0.09	2.38	0.018
ABSS → RFES→ESS (c)	0.68	0.21	3.20	0.001
Sobel Test	Z = 6.97; se = 0.65			0.000

Furthermore, the Sobel test yielded a high Z value (Z = 6.97, *p* < 0.001), reinforcing the statistical significance of the mediation effect (Table 3). These results suggest that individuals with higher adventure behavior seeking tendencies are likely to experience greater satisfaction from outdoor events not only directly, but also indirectly through enhanced flow states during participation.

Results from the bootstrap analysis confirm that recreational flow experience plays a significant mediating role in the relationship between adventure behavior seeking and event satisfaction. The indirect effect for the path from ABSS to RFES to ESS was found to be 0.19, with a bootstrap standard error of 0.03. The 95% confidence interval [0.13, 0.25], which excluded zero, provides strong evidence that the indirect effect is statistically significant (Table 4).

**Table 4:** Confidence intervals

The Mediating Role of T-RFES	Effect	BoostSE	BoostLLCI	BoostULCI
ABSS → RFES→ ESS	0.19	0.03	0.13	0.25

## Discussion

This study investigated the relationships among adventure behavior seeking, recreational flow experience, and event satisfaction in outdoor recreational activities. Grounded in Zuckerman’s Sensation Seeking Theory [29] and Csikszentmihalyi’s flow framework [7], this research developed and empirically tested a comprehensive model that clarifies how individual dispositions and experiential states interact to influence satisfaction in nature-based recreation. Importantly, it advances the literature by confirming the mediating role of recreational flow between adventure behavior seeking and event satisfaction, shedding new light on the psychological mechanisms that drive engagement, enjoyment, and meaningful experiences in outdoor leisure contexts.

Our results demonstrated that adventure behavior seeking significantly and directly predicted event satisfaction, indicating that individuals with a higher disposition toward seeking novelty, challenge, and risk tend to experience greater enjoyment and emotional fulfillment during outdoor events. This finding aligns with prior studies highlighting the hedonic and eudaimonic value of adventure engagement [30, 31]. Adventure-seeking individuals are more likely to embrace uncertainty, physical challenge, and exploration, which not only increases engagement but also enhances perceived competence and accomplishment. In our study, participants who scored higher on the adventure behavior seeking reported higher satisfaction levels, confirming that dispositional motivations alone can meaningfully influence experiential outcomes (H1). This result underscores the importance of considering individual differences in recreational program design, as adventure-oriented participants derive intrinsic reward even in the absence of extrinsic facilitators.

In addition to the direct effects, adventure behavior seeking had a substantial positive effect on recreational flow experience. Participants with greater adventure tendencies reported higher levels of concentration, intrinsic enjoyment, and total immersion in activities, demonstrating that personal dispositions toward novelty and challenge serve as powerful catalysts for optimal psychological engagement. These findings support Zuckerman’s Sensation Seeking Theory [29] which emphasizes that risk-oriented personalities actively pursue experiences that elicit physiological arousal and intense engagement. Moreover, they corroborate Csikszentmihalyi’s [7] flow theory, showing that flow is not merely a situational outcome but is influenced by the interaction between individual traits and environmental conditions. Our findings suggest that those with high adventure behavior seeking scores are more likely to achieve flow states because they actively seek conditions that match their skills with challenges, facilitating deep involvement and absorption in the moment (H2). This reinforces the view that adventure participation is a psychologically complex process, combining dispositional tendencies, motivational drivers, and situational engagement to produce meaningful experiences.

Recreational flow experience itself emerged as a significant predictor of event satisfaction, indicating that participants who achieved high flow reported greater enjoyment, fulfillment, and emotional satisfaction during outdoor leisure activities. The strongest correlation observed was between flow and event satisfaction, highlighting that the quality of engagement is a central determinant of positive outcomes. These results align with prior research demonstrating that flow amplifies intrinsic motivation, engagement, and subjective well-being in leisure contexts [10] Flow provides a psychological state in which participants experience deep focus, a sense of control, and intrinsic reward, reinforcing the idea that engagement quality can magnify satisfaction beyond the mere physical or logistical aspects of outdoor activities. In our study, flow functioned as a mechanism that translated engagement in adventure-based activities into enhanced satisfaction, supporting H3 and highlighting the experiential depth of nature-based recreation. While our findings clearly demonstrate that the quality of psychological immersion, or flow, plays a central role in shaping participants’ satisfaction, it is important to acknowledge that other factors such as the physical intensity, novelty, or skill demands of the activities may also contribute to overall satisfaction. Future research could disentangle the relative contributions of psychological versus physical factors to fully understand the determinants of event satisfaction. Additionally, examining diverse populations, varying outdoor contexts, and different types of adventure-based activities could provide deeper insights into how individual traits and environmental conditions interact to influence both flow experiences and satisfaction outcomes.

The mediation analysis confirmed that recreational flow partially mediates the relationship between adventure behavior seeking and event satisfaction. This indicates that while adventure-seeking tendencies directly contribute to satisfaction, they also enhance satisfaction indirectly by promoting immersive flow experiences. In other words, adventure-prone individuals are more likely to enter states of deep engagement, which in turn amplify their emotional and psychological reward from the activity. These findings are consistent with previous literature emphasizing flow as a key psychological mechanism bridging motivation and experiential outcomes [19, 32, 33]. Our results extend this understanding by demonstrating that flow is not only an outcome of participation but also a mediating process through which dispositional traits translate into higher satisfaction (H4). This partial mediation highlights that both intrinsic disposition and experiential quality are necessary components for optimizing recreational outcomes.

Importantly, the partial mediation observed also highlights that the direct effect of adventure seeking on satisfaction remains statistically significant. This suggests that certain characteristics of adventure-seeking, such as a preference for novelty, risk-taking, and challenge, contribute to satisfaction independently of flow. In other words, while flow facilitates immersive experiences, dispositional motivations alone are sufficient to influence enjoyment and fulfillment in outdoor recreational activities. These findings provide empirical support for the claim that the study deepens the integration of SST and Flow Theory, illustrating how individual traits and experiential states jointly produce meaningful recreational outcomes. This theoretical insight extends prior frameworks by highlighting the mediating role of flow as a mechanism that translates dispositional adventure tendencies into both hedonic and eudaimonic satisfaction, offering a more nuanced understanding of the psychological processes underpinning outdoor leisure.

Beyond the tested hypotheses, the study provides important insights into the psychological and developmental benefits of outdoor adventure participation. Engaging in challenging natural environments has been shown to enhance self-efficacy, resilience, emotional regulation, and identity development [34–36]. The partial mediation observed in our study suggests that while dispositional adventure tendencies facilitate engagement, the quality of the experience, operationalized as flow, is crucial for translating engagement into meaningful psychological and emotional outcomes. This has practical implications for designing outdoor programs that optimize participant immersion by balancing challenge, skill, and autonomy.

Moreover, structured adventure-based activities can support youth development, educational outcomes, and social competencies. Evidence indicates that incorporating outdoor experiential learning into curricula enhances problem-solving abilities, autonomy, collaboration, and resilience [37, 38]. Our findings suggest that fostering flow during such activities can amplify these benefits, offering a pathway for experiential programs to not only provide enjoyment but also support long-term developmental gains. Similarly, therapeutic and preventive interventions may utilize adventure-based engagement to improve mental health outcomes, including stress reduction, emotional regulation, and adaptive coping strategies, with flow serving as a central mechanism facilitating these effects [34, 39].

The study also highlights the importance of contextual factors in shaping flow and satisfaction. Although dispositional tendencies are crucial, environmental features such as perceived safety, challenge level, and novelty influence the likelihood of achieving flow. This aligns with previous studies emphasizing the interplay between individual characteristics and environmental affordances in facilitating immersive engagement [40, 41]. Our results indicate that adventure-seeking participants are most satisfied when the environment allows them to match their skills with challenges, underscoring the need for thoughtful program design that accounts for both participant motivation and contextual conditions.

These contextual factors such as perceived safety, challenge level, and novelty may act as moderators in the adventure seeking–flow–satisfaction model. Theoretically, individuals high in adventure-seeking may achieve optimal flow only when environmental conditions align with their skill and risk preferences. Conversely, in less supportive contexts, the same dispositional tendencies may not fully translate into flow or satisfaction. Including such moderators in the model provides a more nuanced understanding of how personal dispositions interact with environmental affordances to influence outcomes. Future research could empirically test these moderating effects by systematically varying contextual factors across different outdoor settings or participant groups.

Finally, this research contributes to the theoretical understanding of outdoor recreation by integrating dispositional, experiential, and outcome-focused perspectives. Adventure behavior seeking, flow experience, and event satisfaction collectively provide a comprehensive model for understanding how psychological and motivational factors interact to produce meaningful recreational experiences. The findings underscore that outdoor adventure participation is not merely a physical activity but a multidimensional process combining intrinsic motivation, psychological engagement, and experiential quality to foster both hedonic and eudaimonic benefits.

In conclusion, this study demonstrates that adventure behavior seeking influences event satisfaction both directly and indirectly through flow, emphasizing the central role of psychological immersion in nature-based recreation. These results highlight the importance of designing outdoor programs that provide appropriate levels of challenge, novelty, and autonomy, supporting not only enjoyment and satisfaction but also personal growth, resilience, and psychological well-being.

## Theoretical and practical implications

### Theoretical implications

This study contributes theoretically by extending the understanding of outdoor recreation through the integration of dispositional, experiential, and outcome-focused perspectives. By empirically testing the relationships among adventure behavior seeking, recreational flow experience, and event satisfaction, the research clarifies how individual differences in sensation-seeking tendencies translate into psychological engagement and meaningful leisure experiences. Unlike previous research that primarily focuses on participation frequency or physical activity levels, this study positions flow as a central mediating mechanism, demonstrating that immersive experiences amplify the benefits of adventurous behaviors. The findings highlight that adventure-seeking dispositions not only motivate participation but also shape the intensity and quality of engagement, which in turn influences emotional satisfaction and personal growth.

Moreover, the study advances theoretical discussions by demonstrating the multidimensional nature of outdoor adventure participation. Specifically, it integrates psychological constructs (adventure-seeking tendencies), experiential processes (flow), and outcome measures (satisfaction), providing a comprehensive framework for understanding the dynamic interactions that produce both hedonic and eudaimonic benefits. This framework underscores the importance of considering both individual traits and environmental affordances when analyzing leisure experiences, aligning with theories of motivation and positive psychology. By showing that flow partially mediates the link between adventure behavior seeking and satisfaction, the study provides evidence that experiential quality is a key mechanism connecting motivation to meaningful recreational outcomes. This underscores that both dispositional tendencies (as emphasized in Zuckerman’s Sensation Seeking Theory) and experiential processes (as conceptualized in Flow Theory) jointly shape satisfaction. Therefore, our findings empirically support the integration of SST and Flow Theory, highlighting the interplay between intrinsic motivation and optimal psychological engagement in outdoor leisure contexts.

### Practical implications

From a practical perspective, the findings offer valuable guidance for the design and implementation of outdoor recreational programs. Given that adventure behavior seeking significantly predicts flow (H2) and satisfaction (H1), program developers and recreation managers should focus on creating environments that balance challenge and skill levels to optimize engagement. Providing opportunities for novelty, risk, and autonomy can enhance flow states, resulting in higher emotional satisfaction and deeper psychological benefits for participants. The partial mediation of flow (H4) further indicates that designing activities to enhance immersive experiences will translate motivation into meaningful outcomes. In addition, program structures that allow participants to set personal goals, engage in self-directed challenges, and experience progressive skill mastery can foster intrinsic motivation and prolonged participation.

Further, the study underscores the role of psychological immersion in enhancing participant outcomes. Managers should consider environmental features such as safety, accessibility, and varied adventure activities that match different skill levels. Structured yet flexible experiences can accommodate individual differences in adventure-seeking tendencies, ensuring that both novice and highly sensation-seeking participants achieve flow. This approach has implications for educational and youth development programs, therapeutic recreation, and community-based interventions, where outdoor activities are used to promote resilience, problem-solving, and social skills, directly linking the recommendations to our empirical findings. By integrating challenge, novelty, and autonomy into program design, practitioners can enhance not only enjoyment and satisfaction but also psychological growth, self-efficacy, and well-being among participants.

### Limitations and future directions

This study provides preliminary insights into the relationships among adventure behavior seeking, recreational flow experience, and event satisfaction in outdoor recreation, but several limitations must be considered when interpreting the findings. First, the use of convenience sampling from a specific participant group limits the generalizability of the findings. It should be noted that the sample consisted exclusively of Turkish participants, and cultural context may influence the observed relationships. Caution is therefore warranted when generalizing these results to other cultural or national settings. Future research should include more diverse and larger samples across different regions and cultural contexts to to enhance external validity and allow meaningful comparisons across populations and activity types.

Second, although this study focused on core variables, several potentially influential factors were not examined, including perceived safety, environmental conditions, prior outdoor experience, and social support. Incorporating these variables in future research could provide a more comprehensive understanding of the mechanisms that influence flow and satisfaction. Additionally, examining personality traits beyond sensation seeking, motivational orientations, and risk perceptions may help clarify individual differences in recreational engagement. Building on this, the study’s limitations highlight the need to examine contextual factors as potential moderators. Testing how environmental characteristics influence the strength or direction of relationships in the adventure seeking–flow–satisfaction pathway would enhance both theoretical understanding and practical application. Methodologically, future studies could implement experimental, longitudinal, or multi-site designs to rigorously assess these effects, thereby reinforcing the robustness and generalizability of the findings.

It is also important to acknowledge that the external validity of this study is limited due to the characteristics of the sample and the method of data collection. Specifically, a convenience sample was used, and data were collected through online distribution. Therefore, the findings may not be generalizable to the broader population of adults who participate in outdoor recreational activities. Readers should interpret the results with caution, keeping these limitations in mind. Another limitation is the exclusive use of quantitative methods. While this approach enabled hypothesis testing, it may not fully capture participants’ subjective experiences and motivations. Future studies could integrate qualitative methods, such as interviews or focus groups, to provide richer insights into the psychological and contextual factors underlying flow and satisfaction. Finally, future research should consider demographic and experiential moderators such as age, gender, education, skill level, and participation frequency were not systematically examined. Longitudinal designs could explore how adventure behavior seeking and flow contribute to sustained engagement, personal growth, and well-being. Addressing these limitations in future studies will improve the robustness of the findings and inform the development of outdoor programs that promote immersive, enjoyable, and developmentally meaningful recreational experiences.

## Supplementary Information

Supplementary Material 1.

## Data Availability

The dataset supporting the findings of this study is available from the corresponding author upon reasonable request, in accordance with institutional and ethical guidelines.

## Abbreviations

ABSS: Adventure Behavior Seeking Scale
AVE: Average Variance Extracted
CR: Composite Reliability
ESS: Event Satisfaction Scale
M: Mean
RFES: Recreational Flow Experience Scale
SD: Standard Deviation
SPSS: Statistical Package for the Social Sciences
SST: Sensation Seeking Theory
