# Supplementary material for: The mediating role of recreational flow experience in the relationship between adventure behavior seeking and event satisfaction among participants in outdoor leisure activities
Source: BMC Psychol. 2026 Feb 28;14:469. doi: 10.1186/s40359-026-04254-6 (PMC13059190; doi:10.1186/s40359-026-04254-6)
Supplement: Supplementary file 1 — Supplementary Material 1. [file 40359_2026_4254_MOESM1_ESM.docx]

**STROBE Checklist**

| **Item No** | **Reported on Page/Line** |
| --- | --- |
| **Title** | Page 2, Lines 1-25 |
| **Abstract** | Pages 2-3 |
| **Background** | Pages 4-8 |
| **Methods** |  |
| Participants | Page 8, Lines 24-25  Page 9, Lines 1-13 |
| Data collection and ethical procedure | Page 9, Lines 15-25  Page 10, Lines 1-2 |
| Measurements | Page 10, Lines 3-25  Page 11, Lines 1-17 |
| Data Analysis | Page 11, Lines 19-25  Page 12, Lines 1-11 |
| **Findings** |  |
| Descriptives, normality distribution, internal consistency, and convergent validity | Page 12, Lines 13-25  Page 13, Lines 1-15 |
| Conceptual model of research analysis results | Page 13, Lines 16-23  Page 14, Lines 1-19 |
| **Discussion** | Page 14, Lines 21-24  Page 15, Lines 1-25  Page 16, Lines 1-25  Page 17, Lines 1-24  Page 18, Lines 1-25  Page 19, Lines 1-24  Page 20, Lines 1-2 |
| **Theoretical and practical implications** |  |
| Theoretical implications | Page 20, Lines 4-25  Page 21, Lines 1-5 |
| Practical implications | Page 21, Lines 6-25  Page 22, Lines 1-3 |
| Limitations and future directions | Page 22, Lines 5-25  Page 23, Lines 1-16 |
| **Abbreviations** | Page 23, Lines 18-24  Page 24, Lines 1-3 |
| **Ethics approval and consent to participate** | Page 24, Lines 4-10 |
| **Consent for publication** | Page 24, Lines 11-13 |
| **Availability of data and materials** | Page 24, Lines 14-16 |
| **Competing interests** | Page 24, Lines 18 |
| **Funding** | Page 24, Lines 20-21 |
| **Authors’ contributions** | Page 24, Lines 22-23  Page 25, Lines 1-3 |
| **Acknowledgements** | Page 25, Lines 5-6 |
| **References** | Page 24, Lines 8-23  Page 26, Lines 1-23  Page 27, Lines 1-22  Page 28, Lines 1-21  Page 29, Lines 1-23  Page 30, Lines 1-9 |
